# Generic Rules of Mechano-Regulation Combined with Subject Specific Loading Conditions Can Explain Bone Adaptation after THA

**DOI:** 10.1371/journal.pone.0036231

**Published:** 2012-05-02

**Authors:** Tomasz D. Szwedowski, William R. Taylor, Markus O. Heller, Carsten Perka, Michael Müller, Georg N. Duda

**Affiliations:** Julius Wolff Institute (JWI), Center for Musculoskeletal Surgery (CMSC), Center for Sports Science and Sports Medicine Berlin (CSSB), Charité – Universitätsmedizin Berlin, Berlin, Germany; University of Notre Dame, United States of America

## Abstract

Bone adaptation after total hip arthroplasty is associated with the change in internal load environment, and can result in compromised bone stock, which presents a considerable challenge should a revision procedure be required. Under the assumption of a generic mechano-regulatory algorithm for governing bone adaptation, the aim of this study was to understand the contribution of subject specific loading conditions towards explaining the local periprosthetic remodelling variations in patients.

CT scans of 3 consecutive THA patients were obtained and used for the construction of subject specific finite element models using verified musculoskeletal loading and physiological boundary conditions. Using either strain energy density or equivalent strain as mechano-transduction signals, predictions of bone adaptation were compared to DEXA derived BMD changes from 7 days to 12 months post-implantation. Individual changes in BMD of up to 33.6% were observed within the 12 month follow-up period, together with considerable inter-patient variability of up to 26%. Estimates of bone adaptation using equivalent strain and balanced loading conditions led to the best agreement with *in vivo* measured BMD, with RMS errors of only 3.9%, 7.3% and 7.3% for the individual subjects, compared to errors of over 10% when the loading conditions were simplified.

This study provides evidence that subject specific loading conditions and physiological boundary constraints are essential for explaining inter-patient variations in bone adaptation patterns. This improved knowledge of the rules governing the adaptation of bone following THA helps towards understanding the interplay between mechanics and biology for better identifying patients at risk of excessive or problematic periprosthetic bone atrophy.

## Introduction

Periprosthetic bone loss continues to be a burden after total hip arthroplasty (THA) and exhibits substantial inter-patient variability in clinical radiographic measures [1]–[5]. Periprosthetic bone mineral density (BMD) changes are associated with the internal load distribution, where the local bone is thought to adapt to the requirements of loading and function [5]–[8]. Abrupt changes to the internal loading environment, such as after the implantation of a joint endoprosthesis, lead to a short term adaptation of the bone, resulting in periprosthetic bone atrophy in regions that become unloaded. Such bone loss is believed to influence THA longevity by undermining implant fixation and contributing to the risk of aseptic loosening [9], [10]. The resulting compromised bone stock presents a considerable challenge should a revision procedure be required [11]–[13].

Although the mechanisms dictating individual patterns of bone adaptation are thought to be mechanically driven, the rules governing these processes remains unclear. To establish these rules of tissue adaptation, a number of computational models have been developed that aim to predict the changes in periprosthetic bone [14]–[24]. Such predictions of bone adaptation generally rely on models of the interplay between mechanics and biology [25], in which an increase in local loading leads to net bone deposition, while decreased loading results in bone loss, in order to return to customary physiological load levels within the tissue. Previous investigations have predominantly used changes in strain energy density (SED) following THA [9], [14], [22], [26], [27] as a remodelling signal to induce bone mineral changes. The choice of strain energy density was reasoned as being an indirect measure that represents a simplified index for bone gain or bone loss [14], [15]. However, an investigation comparing the adaptation driven by different mechanical remodelling signals did not show strain energy density to be superior against the outcome of animal experiments *in vivo* [17].

In addition to animal experiments, determination of the accuracy and validity of remodelling algorithms in clinical THA cohorts has relied upon comparisons against averaged longitudinal measurements [22], [23]. Such generic comparisons have partially achieved good agreement with RMS errors of approximately 5% [23] against cohort averaged DEXA measurements. However, these comparisons could not account for excessive trochanteric resorption [22] and predictions of less net resorption than occurred *in vivo* was observed in Gruen zones 1 and 2 [23]. Whilst the rules of mechanically regulated bone remodelling in such models have therefore been able to describe the general patterns of periprosthetic adaptation, they fail to explain the mechano-biological aetiology of the individual variations. Such discrepancies are generally thought to be guided by biological adaptive capacity, where it has been widely considered that the genome, proteome and secretome drive the patient specific response. In this study, we want to analyse to what degree mechanical specificity alone can explain the patient specific variations observed. If such individual adaptation is indeed related to subject specific musculoskeletal loading conditions, then next to genetic pre-disposition and molecular biological pathways, the patient specific intrinsic physical constraints [28]–[30] have to be taken into account if biological adaptation processes are to be successfully characterised.

Currently, the mechanisms of tissue adaptation following e.g. an abrupt change to the loading conditions after THA, are not well understood. Under the assumption of a generic mechano-regulatory algorithm for governing bone adaptation, the aim of this study was to understand the contribution of subject specific loading conditions towards explaining the local periprosthetic remodelling variations in patients.

## Methods

### Subjects and DEXA acquisition

Finite element models for predictions of bone adaptation were constructed for 3 consecutive patients who underwent a primary elective total hip arthroplasty, receiving an uncemented Zimmer Alloclassic® stem and Allofit® Pressfit- cup (Zimmer®, Warsaw, IN, USA) from the identical surgical team. The study was approved by the local ethics committee, and after providing written informed consent to participate, each of the three patients (2 female, 1 male, aged 68.7±2.3) received a three joint CT scan (hip, knee, ankle) that included the entire femur, both pre- and post-operatively (GE Centricity, 0.5 mm pixel size, slice thickness of 1 and 5 mm respectively). In addition, radiographic (DEXA) measurements were performed on each patient at 7 days and 12 months after surgery, and changes in BMD were determined within each Gruen zone.

### Finite element models

Pre-operative femoral bone geometry was reconstructed using automated statistical shape modelling techniques (ZIB Amira, Zuse Institute Berlin) [31] with manual correction if necessary. Post-operative femoral geometry, specifically including the location of the resection plane and implant position, was determined from the post-operative CT scan. The triangulated surface reconstructions (intact, post-operative and implant) were converted into NURBS surface representations and anatomic landmarks were determined using the methods of Heller and co-workers (2001) (Geomagic Studio 10, Geomagic Inc., Triangle Park, NC). Virtual implantation and resection of the intact femoral head was performed using Boolean operations on the parametric representations of the femur, implant and resection plane (NX 3.0, Siemens PLN, Köln, DE).

**Figure 1:**
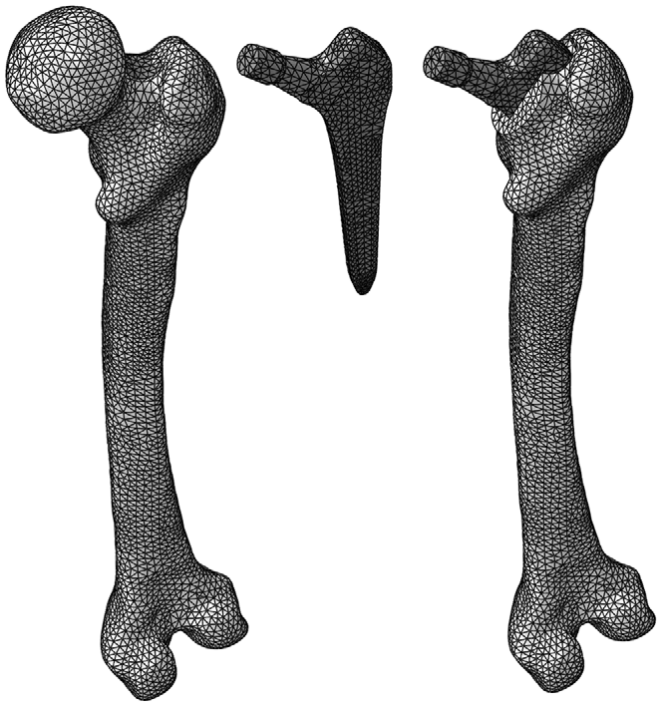
Schematic showing the intact femur (left), implant (centre) and implanted femur (right) meshes, consisting of approximately 80,000 second order tetrahedral elements.

The post-operative femur and implant assembly, that inherently included the anatomy of the intact femur, was then meshed using parabolic tetrahedral finite elements (MSC Patran 2008, MSC Software Corp., Santa Ana, CA) using a global mesh seed of 3.5 mm with a local refinement in areas of high detail (Figure 1). The mesh contained all solids required to construct both the intact and implanted models from the single assembly, allowing mesh congruency in regions common to the intact and implanted femurs, thus facilitating direct mapping of mechanical signals between the models. All models accounted for non-linear geometric effects (Abaqus 6.7-1, Dassault Systèmes, France).

### Material Properties

A calibration phantom (Model #13002 Mindways Software, Inc., CA, USA) was placed under the thigh during all pre-operative CT scans, which allowed transformation of the CT values (in Hounsfeld units) into an apparent bone density. These average local densities were geometrically mapped onto each finite element of the femur model. Elastic modulus was then determined for each element by scaling the apparent density according to an established empirical relationship: E = 15.1 ρ^2.25^ [32]. All materials were modelled as linear elastic isotropic assuming a Poisson's ratio of 0.3.

### Individual Loading and Mechanical Boundary Conditions

**Figure 2:**
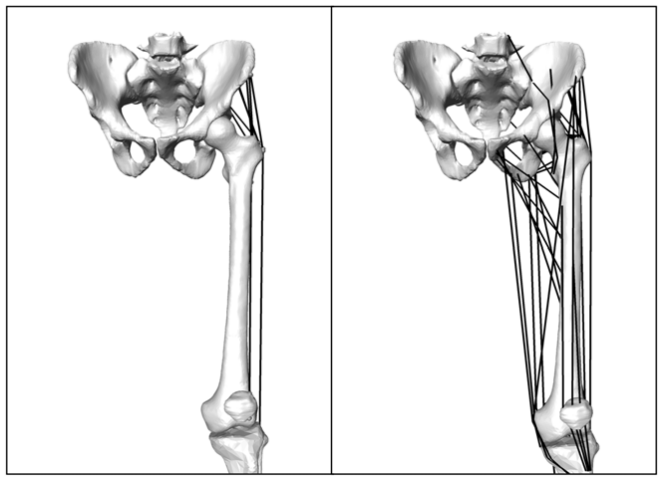
Simplified (left) and complex (right) musculoskeletal models used in the determination of subject specific muscle and joint loading. The simplified loading employs only the musculature that acts over the trochanteric region.

Two sets of different musculoskeletal loading conditions were applied to each patient model and were classified as either simplified or complex. In the simplified loading scenario a reduced set of musculoskeletal loads (Figure 2). was applied to both the pre- and post-operative models according to published data from Heller and co-workers [33]. The musculoskeletal forces were scaled to each patient's body weight and the femur was fully constrained at the mid-diaphysis.

For the complex loading scenario, the intact and implanted hip, knee and ankle centres, femoral neck axis and femoral shaft axis were determined and used to transform all reconstructions into a local femoral coordinate system. Musculoskeletal loading was determined for each patient for both the intact anatomy and the implanted femur configurations using a previously validated musculoskeletal model [34], [35]. This analysis accounted for subject specific anatomy/implantation (femoral antetorsion, caput-collum-diaphyseal angle (CCD) angle, neck length, limb length, limb alignment), as well as muscle origins and insertions (Figure 2). The forces corresponding to maximum joint loading (20% gait cycle) for the intact and implanted anatomies were then applied to the respective models.

### Bone Remodelling Algorithms

**Figure 3:**
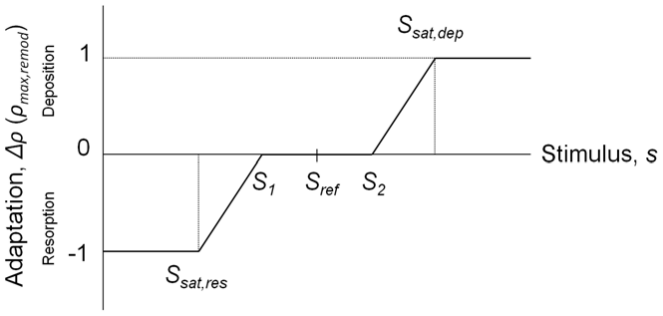
Mechanostat remodelling scheme. Change in the local density of bone for each time step, 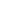, was governed by the local stimulus (*s*) according to the following rules:
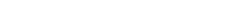


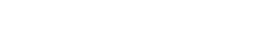


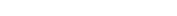


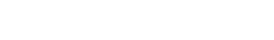


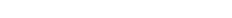
where 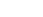 and 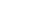 were the stimuli at which deposition and resorption saturation occurred respectively, and *S*_1_ and *S*_2_ defined the limits of the lazy zone. 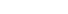 was the maximum rate of change of density for each of the remodelling steps.

Bone remodelling was governed according to a tri-linear curve [9] that possessed a ‘lazy zone’ in which net bone apposition and deposition rates remained in equilibrium, while increased loads stimulated local bone deposition and unloading resulted in local bone resorption (Figure 3). In a first step, the equivalent strain ε_equivalent_ = 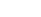 was employed as the mechanical remodelling signal for establishing the reference (intact pre-operative) loading conditions. This signal for the implanted model was then compared with the intact model and the course of remodelling simulated using an iterative adaptive bone remodelling algorithm. The lazy zone (*S*_1_ & *S*_2_) was specified as ±100 µε relative to the intact element-specific equivalent strain [16]. Remodelling proceeded in an iterative fashion, where a reduction in mechanical remodelling signal relative to the intact situation stimulated a decrease in element density and vice versa with a maximum change per iteration (saturation level) when the signal difference was 2000 µε or more. Mechanical remodelling signal deviations of between 100 (*S_sat,res_*) and 2000 µε (*S_sat,dep_*) from the reference signal resulted in a linear change in remodelling to a maximum rate or (saturation) of density change. A parametric study was performed to identify the remodelling rate (saturation) for which 25 iterations successfully reached remodelling equilibrium (all elements < 15% of max remodelling). To compare remodelling signals, a similar iterative model was investigated using strain energy density (SED) to drive the adaptive changes.

### Post-processing

For comparison against DEXA measurements, the bone mineral densities for each finite element were sampled onto a regular grid and projected to simulate a 2D DEXA image. The bone mineral content contained within each Gruen Zone was then computed and normalized against the projected area (area BMD) that included only bone and excluded regions coincident with the implant. The ability of the various models to predict the clinical changes in BMD due to adaptation from the post-op to the 1 year follow-up time point was determined by evaluating the RMS error between the predicted and measured BMD changes for each Gruen zone.

## Results

**Figure 4:**
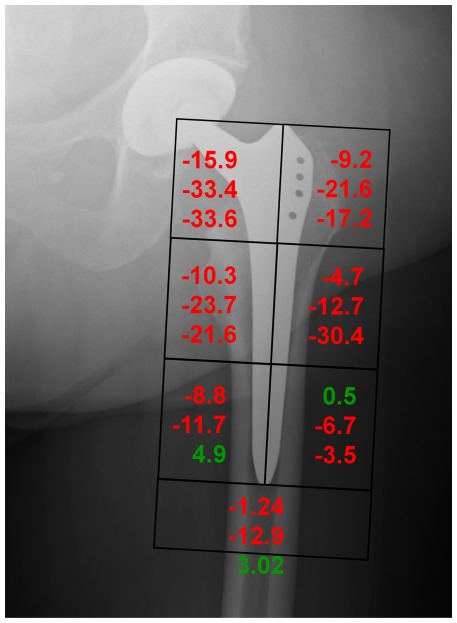
Summary of clinically measured changes in BMD from 7 days to 12 months after surgery. Values are presented for each patient as the percentage change relative to the 7 days postoperative BMD.

Over the course of the investigation, none of the patients experienced postoperative complications. The 3 patients exhibited periprosthetic bone atrophy of −8.1, −18.4 and −16.9% 12 months postoperatively, as measured using DEXA and averaged over 7 Gruen zones. All three patients exhibited atrophy in the most proximal regions (Gruen zones 1 and 7), ranging from −9.2 to −17.2% and −15.9 to −33.6% respectively (Figure 4).

**Figure 5:**
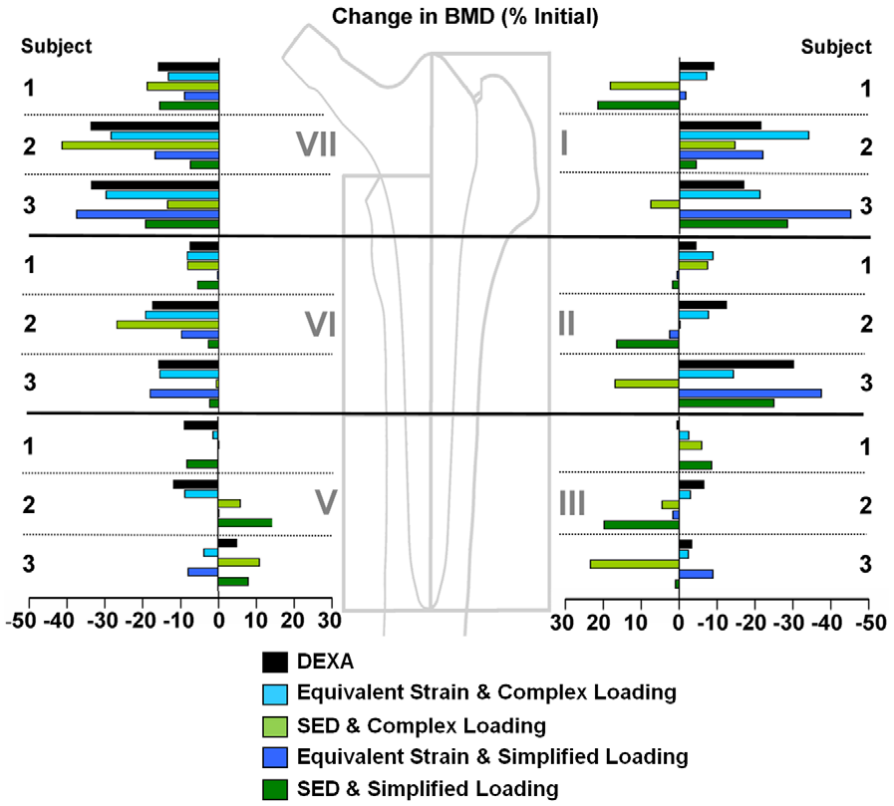
Comparison of change in BMD (% from initial) measured using DEXA against the adaptation signal based predictions of BMD. Also shown is the effect of simplified versus complex musculoskeletal loads depicted as the total change in the BMD of individual Gruen Zones.

The corresponding FE models demonstrated average BMD changes (from 7 days to 12 months after implantation) of −7.5, −18.1 and −15.5% relative to the immediate postoperative BMD for the 3 patients respectively. Individual Gruen zone specific changes measured using DEXA were then compared to the subject specific FEA predictions, and exhibited considerable variation across patients and also as a consequence of loading conditions and mechano-regulation were observed (Figure 5).

For all three subjects, FEA predictions of the bone adaptation within each Gruen Zone demonstrated that mechano-regulation based on equivalent strain and that incorporated the complex musculoskeletal loading conditions (including physiological boundary constraints) resulted in the best agreement with the clinical DEXA measurements (Figure 5). The mean RMS error for the 3 patients was 6.2% while the patient specific RMS errors were 3.9%, 7.3% and 7.3% (Table 1). Adaptation based on equivalent strain with simplified loading conditions, rather than loading conditions that included the full anatomical complexity, reduced the levels of agreement with DEXA measurements to 10.7% overall. The overall best and worst case predictions on a patient and Gruen zone level were: equivalent strain and complex loading (best: 0.4%, worst: 16.0%), equivalent strain with simplified loading (0.4%, 28.1%), SED with complex loading (0.9%, 42.3%), and SED with simplified loading (0.3%, 84.4%) (Figure 5).

**Table 1:** The root mean square (RMS) error (%) values for subject specific comparisons between the 12 month *in vivo* DEXA measurement and model variations that include equivalent strain and strain energy density based remodelling and their combinations with simplified and complete balanced musculoskeletal loading conditions.

RMS error (%)	Subject			
	1	2	3	Mean
Equivalent Strain & Simplified Loading	6.6	13.1	12.4	10.7
Equivalent Strain & Complex Loading	3.9	7.3	7.3	6.2
SED & Simplified Loading	12.6	39.8	17.8	23.4
SED & Complex Loading	12.3	18.7	29.4	20.2

SED based predictions with complex loading had an average RMS error of 20%, which increased to 23.4% under simplified loading conditions. Individually (Table 1): subject 1 exhibited little change in agreement (complex loading; 12.3%, simplified loading; 12.6%), subject 2 showed an overall improvement of 21.1%, while subject 3 exhibited a reduction in agreement with complex loading (−11.6%).

**Figure 6:**
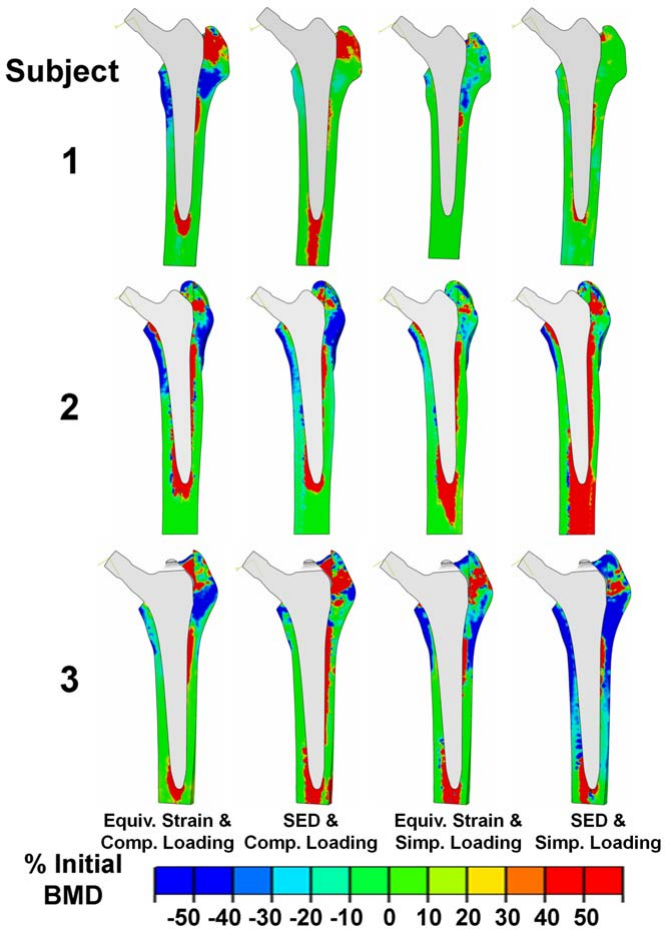
Percentage change in BMD distribution due to adaptation predicted using equivalent strain and strain energy density signals, as well as due to loading conditions, from the implanted to 12 months post-op situation.

In general, the three patient models exhibited unloading in the lower trochanteric region (Gruen zones 1 and 2), and a localized increase in loading directly adjacent to the implant in the proximal lateral region (Gruen zone 1). These patterns correspond to atrophy and bone densification respectively in the trabecular region (Figure 6). Additionally, patterns of unloading of the calcar region (Gruen zone 7) and an increase in loading at the tip of the implant (Gruen zone 4) corresponded to regions of atrophy and densification respectively. Regions of net bone atrophy and deposition exhibited similarities between the two signal types in the complex loading environment, but the application of simplified loading conditions led to more locally varied adaptation patterns.

## Discussion

Bone loss following THA is well documented and frequently associated with changes in the mechanical environment. A detailed understanding of the rules that governing bone adaptation as well as the influence of subject specific factors is, however, lacking. In this study, we assumed that generic mechano-regulatory rules govern bone adaptation and then evaluated the contribution of subject specific anatomy and loading conditions towards explaining the local periprosthetic remodelling variations in 3 patients. The results of this study suggest that while generic rules of mechano-regulation can indeed estimate the broad patterns of periprosthetic bone adaptation observed *in vivo*, knowledge of the subject specific anatomy and loading conditions is indeed required before local variations in bone mineral changes can be discriminated.

This study has demonstrated that considerations regarding balanced musculoskeletal loading conditions [36] that enforce physiological deformation patterns [29], [36] play a critical role for accurate predictions of periprosthetic bone adaptation. It is known that subtle changes to a subject's anatomy can play a critical role the internal loading conditions [37]. It therefore becomes apparent that simplified loading assumptions that have been predominantly employed in periprosthetic femoral remodelling studies, may not capture the complex balanced, subject specific loading conditions [36], and therefore enforce non-physiological deformation constraints [29]. Simplified loading conditions therefore seem limited in their ability to contribute towards understanding the underlying mechanisms that control the subject specific maintenance of bone stock [21].

In our study, 3 patients were observed over 12 months, resulting in average *in vivo* Gruen zone BMD changes of approximately −14.5%, with an RMS error of 6.2%. These results demonstrated that by considering detailed, subject specific loading conditions, the highly variable *in vivo* BMD outcomes of our three patients were all well predicted using our techniques (Figure 4). Importantly, the patient group displayed variations in periprosthetic bone adaptation patterns with average BMD changes ranging between −8.1 and −18.0%, with considerable inter-patient variation between the Gruen zones, and these variations could be explained in the models. Here, the equivalent strain based predictions and complex musculoskeletal loading conditions derived from subject specific analyses were accurate to within the magnitude of error observed in longitudinal DEXA measurements [38]. Although the study by Turner and co-workers [23] has suggested good agreement (5% RMS error) between longitudinal BMD changes and remodelling predictions based on equivalent strain, these predictions were assessed against cohort averaged values of bone remodelling, where local variations between and within individuals become obscured.

This study was able to compare the outcome of equivalent strain or SED mechano-regulation, and specifically how abrupt changes in the stimulus that result from implantation dictate predictions of periprosthetic bone remodelling (Figure 6). The changes in the signals (Figure 5) indicated that an unloading occurred most prominently in: 1. the calcar region (Gruen zone 7) and 2. the proximal lateral regions (Gruen zones 1 and 2), with localized increases adjacent to the implant. These changes were associated with decreases in BMD in the same general locations, reflecting clinical observations [3], [38] with the notable exception in some cases that densification adjacent to the implant occurred in Gruen zone 1. Since these local phenomena appear to be due to boundary effects at the bone-implant interface, more detailed models that incorporate such interactions may be required before a more complete understanding of the local load transfer mechanisms across this interface can be reliably achieved.

The results of this study have demonstrated a better agreement between longitudinal clinical radiographic measurements and computational bone remodelling predictions based on equivalent strain than those based on SED [9], [14], [26], [39] as the mechano-regulatory signal. This result is supported by previous work predicting experimentally observed adaptation patterns in a turkey ulna model [16] that did not find SED to be superior to other mechanical signals. Although these studies have contributed towards instilling confidence in the ‘mechanostat’ model of bone adaptation [40], [41], individual specific patterns and their dependence on musculoskeletal forces, including implantation, have never been demonstrated. Equivalent strain as an alternative to SED was first used by Taylor an co-workers [17] and Turner and co-workers [23] based on work by Stülpner and co-workers [42] and derived from Mikic and Carter [43]. The rationale was that although bone adaptation stimuli could be based on stress or strain, strain was a parameter most likely detectable by bone. Furthermore, in the study of Kerner and co-workers [22], the SED mechanical signal was suggested to be responsible for resorption of the lesser trochanteric region, a result deemed clinically unrealistic. The results presented in this current study indicate that SED consistently invokes a proportionally larger change after implantation and exhibits larger local signal gradients than equivalent strain (Figure 6). This is likely related to the fact that SED is proportional to the square of the strain and therefore more sensitive to the changes in pre- to post-op signal.

For the first time this work considered the effect of physiological boundary conditions that enforce realistic femoral deformation patterns [29] and therefore internal loading conditions on predictions of bone adaptation. Additionally, in this study, the muscle and joint contact forces were taken from a musculoskeletal model [33], [35] that had previously been validated against forces measured *in vivo* using instrumented telemetric hip implants [44]. Musculoskeletal loading in this study was adapted to the patients' geometry, where the musculoskeletal force predictions took into account the hip joint anatomy both before and after implantation. Patient specific gait patterns, however, were not included in this study, where kinematics were assumed to be the same both before and after implantation. The role of including subject specific gait patterns and external ground reaction forces might alter the characteristics of load estimations. Although not available in this study, one elegant approach for differentiating the impact of periprosthetic stress-shielding from global changes in limb loading, could be to normalise the proximal BMD to a reference BMD taken at a location well distal to the stem tip (sufficiently below Gruen Zone IV) pre- and post-operatively [45].

This investigation was able to achieve good agreement with DEXA BMD measurements despite certain limitations. DEXA can only produce a two dimensional projection that inherently reduces the amount of information available regarding the distribution of BMD. The third dimension is partially preserved in the operation of planar summation, but the spatial distribution is partially obscured. DEXA measurements also obscure information in regions that are coincident with the implant, and simplification into individual Gruen zone values further reduces the local specificity. However, in this study, DEXA allowed a quantitative and reproducible comparison for determining the adaptation of bone at multiple locations for longitudinal assessment of BMD in these subjects.

In summary, this study has demonstrated in three THA patients that under the assumption of generic bone adaptation rules, the variability in bone remodelling patterns between individuals can only be predicted when subject specific anatomy and loading conditions are taken into consideration. Additionally, this study has shown that mechano-regulation driven by an equivalent strain signal can produce good estimations of bone tissue adaptation. Here, the high level of agreement against clinical measurements using these techniques establishes a foundation for examining the variation in individual BMD adaptation patterns. This improved knowledge of the rules governing the adaptation of bone following the abrupt mechanical changes that occur due to the implantation of a prosthesis helps towards understanding the interplay between mechanics and biology for better identification of patients at risk of excessive or problematic periprosthetic bone atrophy.
